# Livestock Slurry and Sustainable Pasture Management: Microbial Roles, Environmental Impacts, and Regulatory Perspectives in Ireland and Europe

**DOI:** 10.3390/microorganisms13040788

**Published:** 2025-03-29

**Authors:** Mariana Juca Silva, Ismin Zainol, João Rui Tanoeiro, Aline Sitowski, Ian Major, Emma J. Murphy, Gustavo Waltzer Fehrenbach

**Affiliations:** 1 Bioengineering Organ-on-Chip Research Group (BOC), Centre for Applied Bioscience Research, Limerick Campus, Technological University of the Shannon, V94 EC5T Limerick, Ireland; mariana.jucasilva@tus.ie (M.J.S.); emma.murphy@tus.ie (E.J.M.); 2 Department of Engineering, Polymer, Recycling, Industrial, Sustainability and Manufacturing Research Institute (PRISM), Athlone Campus, Technological University of the Shannon, N37 HD68 Athlone, Ireland; isminzainol@gmail.com (I.Z.); a00304102@student.tus.ie (J.R.T.); ian.major@tus.ie (I.M.); 3 Post-Graduation Program in Biology of Fungi, Algae, and Plants, Department of Biology Sciences, Federal University of Santa Catarina, Florianopolis 88040-900, Brazil; aline_sit@hotmail.com

**Keywords:** antimicrobial resistance, carbon emissions, microorganisms, nutrient leaching, slurry fertilization

## Abstract

Pastures serve as the primary source of grass and forage plants for grazing livestock, requiring adequate nutrient input to sustain growth and soil fertility. Slurry from the livestock industry is widely utilized as a sustainable and cost-effective alternative to chemical fertilizers. Microorganisms within the slurry–pasture system are essential for breaking down organic matter, facilitating nutrient cycling, and improving soil health. However, mismanagement or inefficient microbial decomposition can lead to significant issues, such as nutrient leaching into water bodies, causing eutrophication, antimicrobial resistance, and reduced nutrient availability in pastures, which, in turn, may negatively impact livestock productivity. Thus, this paper investigates the composition and benefits of livestock slurry in pasture management, highlights microbial roles in nutrient cycling, and evaluates regulatory frameworks in Ireland and Europe. Additionally, it examines the environmental risks associated with improper slurry application, providing insights to support sustainable management practices.

## 1. Introduction

The agri-food industry is an essential sector of Irish socio-economy, responsible for 6.5% of national employment and gross value added at factor cost of EUR 17.3 billion in 2022. Dairy products and beef are the main merchandise products exported, relying on pasture quality for optimal feeding and product excellence [1]. This reliance is demonstrated by the increase of 34.7% in the input price index of agricultural products due to a 42.9% rise in energy prices and 121.3% rise in fertilizers in 2022 [2]. The use of livestock slurry generated in dairy production and beef is a strategy for nutrient supply and production costs reduction that has been employed in Ireland and several other countries. Livestock slurry is a valuable resource for grass and pasture production, and is considered a source of essential nutrients such as carbon (C), nitrogen (N), phosphorus (P), potassium (K), and micronutrients. However, this resource must be handled properly to reduce the environmental risks associated with its application in pastures and soil. This was presented in the study by Zhu et al. [3], who investigated the profile of human bacterial pathogens, virulence factor genes, and antibiotic resistance genes in soils amended with pig manure, chicken manure, cow dung, silkworm excrement, background soil, and chemical fertilizer. The authors identified pathogenicity in 1.33% of total bacteria screened, and abundant and diverse virulence and antibiotic resistance genes. Pig manure and silkworm excrement increased higher microbiological risks in soil.

In the process of nutrient recycling and risk mitigation, microorganisms play an important role [4] Bacteria and fungi are primarily responsible for breaking down the complex organic compounds found in livestock slurry, such as proteins, carbohydrates, and fats. Bacteria, including aerobic and anaerobic species, initiate the breakdown of these compounds into simple molecules like amino acids, sugars, and fatty acids [4,5]. While fungi contribute by decomposing more complex molecules organic materials, particularly fibrous substances like lignin and cellulose. The adaptive behavior of microorganisms is an important factor in survivability, allowing their use and application in different conditions [6,7]. Microorganisms are also active players in climate change. They produce and consume the three dominant greenhouse gases that are responsible for 98% of the increased warming: carbon dioxide, methane, and nitrous oxide [8]. In aerobic environments such as slurry fertilized fields, soil microbial communities regulate the reflux of these gases and impact atmospheric composition. The interplay between microorganisms and nutrient cycling is crucial and its disbalance contributes to environmental pollution, with nitrate leaching and eutrophication posing a substantial risk in aquatic systems [9].

To maximize the potential of slurry as a nutrient source, employing microbial strategies is essential. These strategies can include the use of tailored microbial inoculants to accelerate the decomposition process and improve nutrient release. By harnessing the natural abilities of microorganisms, farmers can enhance the quality and uptake of nutrients, leading to better plant growth, reduced emissions, and minimized nutrient leaching [10].

The combined use of microbial consortia and slurry significantly improves plant growth, as seen in previous studies showing increased root and shoot dry weights and higher foliar nutrient levels. This combination also enhances soil chemical properties and nutrient uptake by plants [11]. Also, slurry application can positively affect the abundance of nitrogen cycle microbial guilds, such as nitrifiers and denitrifiers, which play crucial roles in nutrient dynamics and availability [12]. In addition, it is possible to significantly reduce methane emissions during slurry storage through the addition of effective microorganisms and carbon sources to slurry. This is achieved through microbial anaerobic respiration and self-acidification, which lower slurry pH and inhibit methane production [13]. Biogas slurry application increases soil organic carbon (SOC) stocks and enhances the complexity of soil bacterial communities involved in SOC cycling, suggesting that biogas slurry can be beneficial for long-term soil health and carbon sequestration [14].

The need for more sustainable and efficient technologies in agriculture is widely supported by farmers, society, and policymakers. Ireland’s agricultural regulations aim to protect water quality, reduce greenhouse gas emissions, and promote sustainable farming, ensuring that agriculture aligns with environmental protection efforts. Additionally, Ireland’s Climate Action Plan aims for a climate-resilient and sustainable economy by 2050, emphasizing the agricultural sector’s role in reducing emissions through improved slurry management and sustainable farming techniques [15,16]. The development and adoption of improved agricultural practices, however, can be influenced by various factors, including economic, social, and political considerations. A study by Artner-Nehls et al. [17] analyzed 4227 articles published between 2010 and 2020 in three specialized farming magazines in Germany and observed a shift in coverage of slurry and water management, showing reduced attention to innovations for water-conserving slurry management and an increase in discussions reflecting the broader policy debates.

To investigate the role of livestock slurry in sustainable pasture management, this review paper discusses key aspects of its application in pasture fertilization, elucidating its nutrient composition, microbial contributions to nutrient assimilation and cycling, environmental risks, the role of microorganisms in cattle nutrition, best practices for agricultural use, policies promoting sustainable slurry management, and future directions and research needs. Understanding the agricultural challenges in Ireland and wider Europe in relation to use of livestock slurry, this manuscript addresses several points of interest for local development, including the discussion of policies, regulations, and data from national agricultural agencies. Proper pasture management and regulatory oversights are essential for maximizing the benefits of livestock slurry while promoting sustainable agricultural practices.

## 2. Livestock Slurry

Irish agriculture relies heavily on livestock production, which, in turn, produces large volumes of nutrient-rich by-products, particularly cattle slurry [18]. This product is defined as a semi-fluid and heterogeneous mixture comprising animal manure, water, and traces of bedding materials and leftover feed [19,20]. Recent data from the Agriculture and Food Development Authority in Ireland [21] estimate that Irish farms produce approximately 40 million tons of animal manure annually.

**Figure 1:**
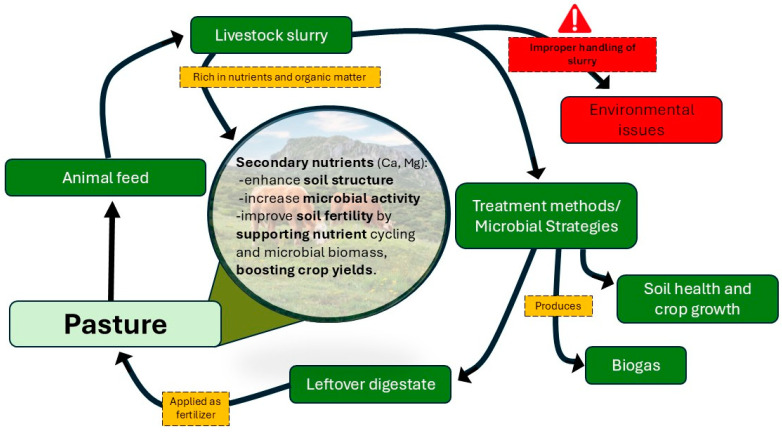
Livestock slurry, its utilization patterns and environmental issues.

The nutrient profile of livestock slurry is rich and diverse, containing essential macronutrients such as N, P, and K, which are vital for plant growth. Because of its nutrient-enriched profile, slurry is recognized as a valuable organic fertilizer [22]. These nutrients are present in both organic and inorganic forms, providing both immediate and slow-release benefits to crops. Slurry also contains secondary nutrients like calcium (Ca) and magnesium (Mg), as well as micronutrients such as zinc (Zn), copper (Cu), and manganese (Mn), which support soil health and enhance crop yields (Figure 1). The combination of these nutrients improves soil structure, increases microbial activity, and contributes to overall soil fertility [20].

The composition of cattle slurry varies significantly due to factors such as farm management practices, animal diet, housing systems, and meteorological conditions. The nutrient concentration is closely linked to the slurry’s dry matter content, with higher dry matter levels corresponding to increased nutrient availability [13]. Cattle slurry typically has a dry matter of 6%, with available nutrient values of 1.0 kg/m^3^ of N (9 units per 1000 gallons), 0.5 kg/m^3^ of P (5 units per 1000 gallons), and 3.5 kg/m^3^ of K (32 units per 1000 gallons) [14].

A better understanding of the factors influencing nutrient composition is essential for optimizing slurry use in agriculture while minimizing environmental impacts [15]. Proper storage preserves N levels, thereby enhancing the nutritional value of the slurry. Adequate storage capacity allows for controlled application at the optimal time when crops need nutrients. Additionally, the timing of application influences N availability, with spring being the most effective season for maximizing nitrogen recovery. To prevent nutrient dilution, losses, and environmental contamination, it should be applied when no heavy rainfall is forecast, and should adhere to distance regulations. Specifically, slurry should maintain a minimal distance of 5 m from surface waters (extending to 10 m for the first two and last two weeks of the spreading season), 10 m from surface waters where the slope towards the water exceeds 10%, 15 m from exposed cavernous or karst features such as swallow holes and exposed rock, 20 m from a lake shoreline, and 25–200 m from a water abstraction point for human consumption [23].

Historically, manure has been used as a beneficial soil enhancer, with its application dating back to ancient agricultural practices. However, with the modernization of farming in the 1940s, chemical fertilizers became the preferred means of boosting crop productivity. In response to growing concerns over the environmental impact of synthetic fertilizers, the European Union implemented the Fertilizing Products Regulation of 2019, which expanded the use of recovered and bio-based fertilizers. This regulatory shift aligns with the EU Circular Economy Package of 2015, emphasizing sustainability within the agro-industrial food supply chain [24].

Unlike chemical fertilizers, slurry application supports agricultural productivity in a cost-efficient and eco-friendly manner [25]. It improves soil organic matter, structure, moisture retention, and biological activity while increasing nutrient availability [26]. These benefits enhance pasture productivity and support sustainable agricultural practices (Figure 1). Additionally, slurry can increase microbial biomass and activity, leading to enhanced nutrient cycling [27].

Slurry is rich in N compounds, particularly in ammonium (NH_4_^+^). When applied to soil, it alters the composition of microbial communities enhancing bacterial and fungal populations, with a dominance of bacteria [27,28]. Tang et al. [14] reported that biogas slurry led to more complex bacterial interactions compared to chemical fertilizers, likely due to increased soil fertility. In contrast, fungal networks became simpler, possibly due to bacterial–fungal competition. Moreover, biogas slurry treatment has been shown to stimulate the release of microorganisms specialized in humus decomposition, along with lignocellulolytic enzymes, which are essential for breaking down lignin and cellulose in plant residues. The increased enzymatic activity accelerates the degradation of recalcitrant organic material, promoting soil carbon cycling and improving overall soil health [29].

In contrast to other organic fertilizers like compost or solid manure, slurry has higher liquidity, which allows for a more uniform application and rapid nutrient absorption. This application aligns with goals for agricultural productivity and environmental conservation. The use of slurry enhances soil microbial activity, supports crop growth, and may contribute to the suppression of pests and diseases [30].

Currently, the main methods for treating livestock manure include direct field application, aerobic composting, anaerobic digestion, solid–liquid separation, and chemical treatments. These methods aim to increase the efficiency of nutrient recovery, minimize environmental contamination, and enhance the resource value of manure. Digestion methods (aerobic/anaerobic) can be used for the removal of N and organic loads from animal waste [31]. Anaerobic treatment is mostly popular in Europe due to its benefits on biogas production, which is used for electricity and heat [19].

During anaerobic treatment, microorganisms break down organic matter, leading to the formation of acetic acid, which is then utilized by methanogens to produce biogas (CH_4_). The by-product of this process is called digestate (or biogas slurry), a nutrient-rich slurry that can be used as a fertilizer which may require additional processing before it can be efficiently applied to fields. Biogas slurry has been shown to regulate the structure of soil bacterial communities and enhance the diversity of soil fungal communities [19,28,31].

## 3. Pasture Culture and Its Importance to Livestock Industry

Pasture contributes substantially to Irish culture for grazing livestock, serving as the backbone for the growth and success of the agriculture industry. Ireland’s agricultural landscapes are mostly characterized by an integrated crop–livestock system (ICLS). This system incorporates crop residues into animal feed, provides a sustainable model for land use, and supports biodiversity. Pasture culture plays a crucial role in the livestock industry in Europe, providing a sustainable and biodiversity-friendly approach to animal husbandry. Proper pastureland management is crucial for providing a feed base for livestock and quality food production [32], as European pasture-based livestock farming plays a crucial role, especially in milk and meat production, while providing essential ecosystem services such as nutrient cycling and C sequestration.

Despite extensive research regarding grassland management and animal production, the diverse and variable nature of grasslands makes it challenging to establish universal guidelines for each specific condition [33,34]. Considering this, the establishment of databases integrates knowledge and organizes data, facilitating data-informed decision making. To address this challenge, the Agriculture and Food Development Authority (Teagasc) developed methods to improve animal performance from grazed pastures, including PastureBase Ireland (PBI) and the improvement of the nutritive value of pasture [35]. More specifically, PBI is a web-based grassland management application designed by Teagasc to compile farm data to help Irish dairy, beef, and sheep farmers to determine appropriate actions around grassland. It also supports authorities by capturing data for benchmarking and research. This tool has a wide array of uses, making it essential for improving productivity and sustainability in pasture-based agriculture [36]. Additionally, sustainable practices can improve the nutritive value of pastures and impact livestock performance. For instance, the inclusion of white clover in perennial ryegrass (PRG) swards is shown to increase the milk production efficiency of lactating dairy cows and reduce the amount of chemical N fertilizer required (−100 kg of nitrogen/ha) (+48 kg of milk solids/cow). This is possible since white clover can offer an alternative source of N, providing biologically fixed N through its symbiotic relationship with *Rhizobium* bacteria [37]. Furthermore, the optimization of herbage mass swards is also essential to improve the nutritional value of the pastures and leads to increased milk production efficiency, dry matter intake, and a reduction in methane emissions [35].

**Figure 2:**
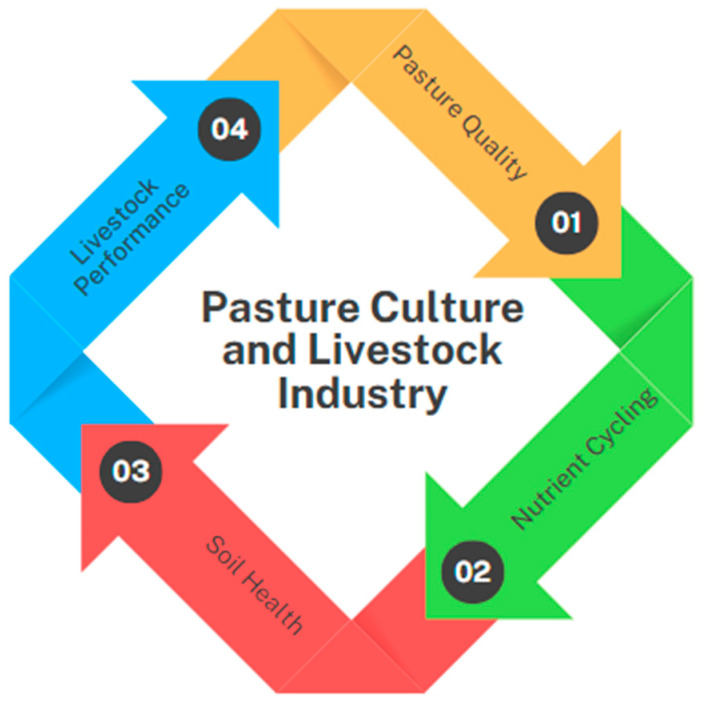
Interconnections of pasture culture with livestock industry productivity.

Nutrient cycling is also a crucial concept in soil fertility in pasture ecosystems because it replenishes nutrients and sustains plant growth, increasing crop productivity. Clearly, many of the nutrients consumed by grazing animals are returned to the environment in feces and urine. For instance, grazing livestock returns approximately 60% of P through manure and 90% of K through urine back to the soil [38]. N, P, and K are critical for pasture biomass and nutritional content. Pasture management practices influence the transformations occurring within these cycles, influencing the productivity of crops and providing sustainable pasture-based livestock production systems (Figure 2) [39].

N is related to the synthesis of essential biomolecules in plants which participate in different stages of photosynthesis and growth. P, a key component of soil fertility, is critical for crop development and plant metabolism. K, in contrast, regulates water flow, enzymatic activation, opening and closing stomata, and transporting carbohydrates [40]. Additionally, it is imperative to emphasize that carbon is also essential for pastures, though it operates differently. It is part of organic soil matter and is responsible for soil microbiome and structural support [41].

Ireland follows the European Union’s Nitrates Directive, which accounts for codes of good agricultural practices aiming to prevent and reduce water pollution from nitrates. Following this regulation, farmers in Ireland are obliged to ensure that the total of N from organic manure applied to their lands does not exceed 170 kg of N/ha/year. Farmers can apply for a nitrate’s derogation where the 170 kg N/ha limit will be exceeded by up to a maximum of 250 kg N/ha/year [42,43]. This is crucial since high concentrations of N from agricultural sources have been found by the European Commission to be one of the main reasons for water pollution in Europe [44].

Nitrates and other N compounds from manure and fertilizers enter groundwater through leaching and reach surface water through runoff from agricultural fields. Furthermore, elevated levels of N as well as P in waters are deeply associated with eutrophication [44]. As such, maintaining the C:N:P:K ratio is indispensable to increase crop yield, as well as promoting the use of sustainable farming practices. A balanced nutrient ratio increases the productivity of agroecosystems by enhancing nutrient cycling through grazing animals and minimizes environmental impacts. This, in turn, leads to a nutrient-dense foliage for grazing animals, and consequently higher quality and production of livestock-based products [45]. Effective pasture management not only supports livestock productivity, but also maintains soil health through balanced nutrient cycling, ensuring long-term sustainability.

## 4. The Role of Microorganisms in Nutrient Assimilation from Slurry-Fertilized Pastures

As primary life forms in soil systems, microorganisms are the main participant in the mineralization of organic matter, nutrient cycling, and even driving climate change [4,5]. Nutrient cycling consists of several interconnected processes that allow the continuous movement, transformation, and recycling of nutrients such as C, N, P, and S continuously within ecosystems [6,7]. These processes transform nutrients into different chemical forms, ensuring their availability to living organisms and returning them to the environment in a sustainable manner [6]. Although nutrient cycling never truly stops, it may be influenced by biological activity (e.g., the variation in microbes in the rhizosphere), environmental conditions (e.g., temperature fluctuation or moisture levels), and seasonal changes (e.g., warmer or wetter months) [6,46]. Microbial metabolic processes regulate the cycling of these key nutrients, with specialized microorganisms driving each stage of the cycle [47,48].

Daily variations may be caused by the length of sunlight on a particular day, such as photosynthesis and respiration rate [6]. Seasonal variation was reported where the microbial activity is generally higher during warmer and wetter months, compared to colder and drier months where the cycle continues at a slower pace [49]. Depending on the process, some are rapid in an oxygenated environment, such as decomposition and nitrification, while some require low-oxygen (anaerobic) conditions, such as the denitrification process [46]. In natural ecosystems, most nutrients (e.g., C, N, P, and S) are bound in organic molecules, and are therefore less bioavailable for plants [7]. To access these nutrients, plants are dependent on soil microbes which possess the metabolic machinery to depolymerize and mineralize organic forms of C, N, P, and S into inorganic forms, including ionic species such as ammonium (NH_4_^+^), nitrate (NO_3_^−^), phosphate, and sulfate (SO_4_^2−^), which are the preferred nutrient forms for plants [7]. There are over 80 known species of microorganisms that play a role in nutrient cycling which can be divided into bacteria, fungi, archaea, protozoa, algae, and cyanobacteria [47].

Bacteria are some of the most versatile and significant contributors to nutrient cycling, playing key roles in various biogeochemical processes [50]. There are various bacteria groups divided according to specific cycle for C (heterotrophic bacteria), N (fixing, nitrifying, and denitrifying bacteria), P (phosphate solubilizing-bacteria) and S (sulfur-oxidizing and -reducing bacteria) [48,49]. Fungi are crucial decomposers, particularly in plant materials [51]. Archae are less known than bacteria but a significant player in nutrient cycling, especially in extreme environments [51,52]. Protozoa are single-cell eukaryotic organisms that contribute to nutrient cycling indirectly by feeding on bacteria and other microorganisms [50]. Algae and cyanobacteria are primary producers in aquatic and some terrestrial ecosystems, playing key roles in C and N cycling [50,53,54]. The classification of the type of microorganisms and their specific roles in nutrient cycling are summarized in Table 1.

Microorganisms are essential drivers of nutrient cycling, influencing soil fertility and ecosystem stability through diverse biochemical processes. The microbial role, mechanism of key processes, and impact on the plants in each individual nutrient cycle (C, N, P, and S) are discussed in the following sections.

### 4.1. Specific Functions of Microorganisms in the Carbon (C) Cycle

Microorganisms play a crucial role in the C cycle in processes that can influence plants, either directly or indirectly [49]. Microbial activity facilitates the breakdown, transformation, and storage of C in various forms to ensure that C is efficiently recycled to support plant growth and ecosystem stability [61]. The microbial activity involved in facilitating the C cycle processes include C fixation, decomposition, humus formation, symbiotic associations, methane production and oxidation, sequestration, rhizosphere cycling, and respiration [49,61]. These activities are defined and summarized in Table 2.

### 4.2. Specific Functions of Microorganisms in the Nitrogen (N) Cycle

The N cycle is a complex series of processes by which N moves between the atmosphere, soil, water, and living organisms [50]. It involves several key transformations carried out by various types of microorganisms that convert N into different chemical forms [65]. Plants require large amounts of N because it is required to synthesize fundamental biological molecules, such as nucleotides, amino acids, and proteins [66] . N not only regulates soil physicochemical property and enzyme activity, but also exerts a profound influence on the proliferation diversity, community composition, relative abundance, and metabolic function of soil microorganisms [54]. The key microbial activities facilitating the N cycle are N fixation, nitrification, ammonification, denitrification, and regulations of anaerobic conditions [53,54,65]. The role of the key microorganisms and the impact of these processes on this cycle are summarized in Table 3.

### 4.3. Specific Functions of Microorganisms in the Phosphorus (P) Cycle

P is essential for plants for its involvement in energy transfer (via ATP) in photosynthesis and respiration processes, and it is a key element for biosynthesis of DNA and RNA and a crucial nutrient in the formation of cell membranes [70]. Since P is often present in insoluble or unavailable forms in soils, microorganisms help mobilize, transform, and make P available for plant uptake [49,70]. The microbial activity that facilitates the cycling of P includes solubilization, mineralization, mycorrhizal associations, organic P cycling, immobilization, and biofilm formation and transport [5,43,54]. The microbial activity, microorganisms, mechanisms, and impacts on the plants are summarized in Table 4.

### 4.4. Specific Functions of Microorganisms in the Sulfur (S) Cycle

S is an essential nutrient for plants that is required for synthesizing amino acids (e.g., cysteine, methionine), vitamins, and enzymes [73]. Microbial activities ensure S is cycled between its different states, maintaining its availability to not only sustain plant health, but also contribute to the broader ecological stability and aquatic ecosystems [74]. The microbial activities that facilitate S cycling include mineralization, oxidation, reduction, immobilization, mycorrhizal uptake, and volatilization. The microbial roles in these activities, their mechanisms, and impacts on plants are summarized in Table 5.

## 5. Environmental Risks Related to Usage of Livestock Slurry in Pasture Fertilization

**Figure 3:**
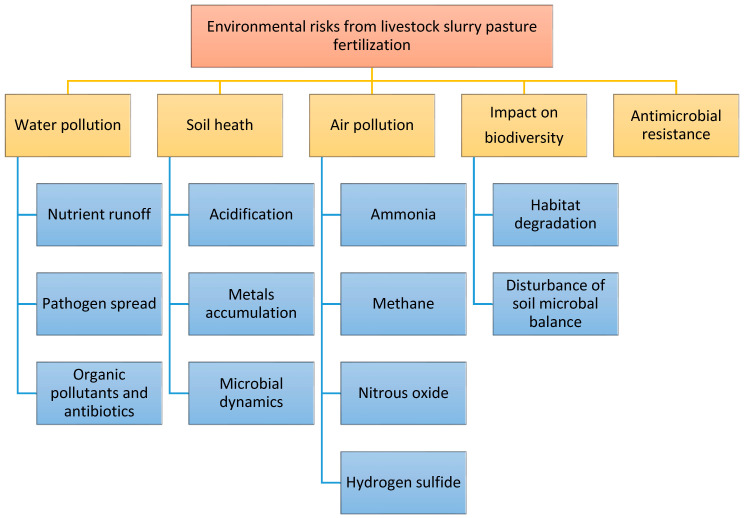
Key environmental risks from pasture fertilization with livestock slurry.

Livestock slurry can also pose environmental risks if not managed carefully. Agriculture systems are known for their complex interactions with the environment. The objective of this section is to understand the effects of livestock slurry in pasture fertilization to identify alternatives and strategies to reduce its environmental impact. These risks are not only exclusive to Irish farmers, but have been reported worldwide. The key environmental risks from pasture fertilization with livestock slurry are presented in Figure 3 and discussed in the following subsections.

### 5.1. Water Pollution

Water pollution happens multidirectionally with the transfer of contaminants, nutrients, pathogens, and organic pollutants from slurry to water bodies such as rivers, lakes, streams, and seas [76]. Nutrient runoff is characterized by the mass transfer of P, N, and other nutrients to surface or subsurface waters via direct discharges and groundwater discharge [77]. This event is highly dependent on weather conditions, the level of nutrients in slurry, slurry’s physical properties (e.g., water, particle size), soil permeability, and application methods. The high input of nutrients to water bodies lead to eutrophication and algal blooms, ecosystem imbalance, and oxygen depletion. The nutrient loss is not only a concern from the environmental perspective, but also economically, as farm yield and nutrient content in pasture might be lower than necessary for plant development.

To increase farm production, it is necessary to assess the application methods available, the conditions, and several other aspects, including slurry composition, weather conditions, and soil characteristics. For example, Johnson et al. [78] conducted a 2-year study with 10 × 13 m^2^ plots treated with dairy slurry followed by a single rainfall episode to evaluate the effects of different slurry application methods on phosphorus runoff. The authors reported variation between years and treatments, with aeration yielding lower phosphorus losses and lower runoff from reduced tillage systems treated with manure slurry. In a similar attempt to improve precision and irrigation dosage of slurry, Jiang et al. [79] reported the re-designing and modeling of traditional connectors, based on factors such as soil and humidity, improving the slurry mixture and application precision, which might minimize pollution to soil and environment.

The application of livestock slurry on farms poses a significant risk for the spread of pathogens, including *Escherichia coli* O157:H7, *Salmonella* spp., and *Cryptosporidium parvum*, which are commonly found in animal feces and can persist in the environment depending on factors like temperature, moisture, and pH [80]. These pathogens can contaminate water through surface runoff during rainfall, infiltrate groundwater in regions with sandy soils or shallow aquifers, and be transmitted to crops or pastures grazed by livestock [80,81]. Additionally, aerosols generated during slurry application can further spread these microorganisms. Human exposure to contaminated water or food can result in gastrointestinal illnesses, while reinfection cycles in livestock are possible, perpetuating disease risks. The presence of antibiotic residues in slurry exacerbates the issue by fostering antimicrobial resistance [82].

The application of livestock slurry to agricultural fields introduces various antibiotics and their metabolites into the environment, raising concerns about ecological impacts and the development of antimicrobial resistance. Commonly used antibiotics in livestock farming include tetracyclines, sulfonamides, and macrolides [83]. Tetracyclines, such as oxytetracycline and chlortetracycline, are frequently administered to cattle, swine, and poultry to treat respiratory and gastrointestinal infections. Sulfonamides, including sulfadimethoxine and sulfamethazine, are utilized for their broad-spectrum antimicrobial properties. Macrolides, like tylosin, are employed to manage respiratory diseases in livestock [83].

In a study to assess the occurrence of antibiotics in free stall dairy farms with manured forage fields, Watanabe et al. [84] reported the presence of 10 antibiotic compounds in manure lagoons, including tetracyclines and trimethoprim. Of these 10 compounds, sulfamethazine and lincomycin were found in a shallow groundwater downgradient from the lagoons, while antibiotics were sporadically detected in surface samples from fields with manure application, but not in subjacent sandy soils. Sulfamethazine and sulfadimethoxine were detected at highly attenuated levels in shallow groundwater near to irrigation points assessing the occurrence of antibiotics. Effective slurry management is crucial to mitigating water pollution, pathogen spread, and antimicrobial resistance, ensuring both environmental and agricultural sustainability.

### 5.2. Soil Health

The use of livestock slurry as a soil and pasture fertilizer has gained popularity due to its cost-effectiveness and nutrient-rich composition. While it provides essential nutrients, such as N, P, and K, its application also has complex effects on soil health. These effects include soil acidification, metal accumulation, and changes in microbial dynamics, which can have both beneficial and adverse consequences depending on management practices.

Livestock slurry is a valuable source of nutrients that can enhance soil fertility and microbial activity. The organic matter in slurry serves as a substrate for microorganisms, stimulating microbial biomass and enzyme activities that are critical for nutrient cycling. Increased microbial activity can improve soil structure, nutrient availability, and pasture productivity [85]. In a 5-year study conducted by Feng et al. [28] assessing the long-term impact on soil nutrients and microbial communities in a rotation system of annual ryegrass–silage maize, the long-term application of biogas slurry significantly increased the soil organic C, total N, available K, and available P, neutralized the soil pH, and contributed to the regulation of the soil’s microbial community structure. However, excessive slurry application can disrupt this balance and lead to the proliferation of pathogenic organisms, which can harm soil health and the broader ecosystem. The frequent application can also contribute to soil acidification, particularly when ammonium-based compounds are present. Ammonium undergoes nitrification in the soil, producing hydrogen ions that lower soil pH. Acidification can reduce the availability of key nutrients such asP and molybdenum, while increasing the solubility of potentially toxic elements like aluminum and manganese. Consequently, long-term slurry use without pH correction may degrade soil health and reduce pasture productivity [86].

Trace amounts of metals such as copper (Cu), zinc (Zn), and cadmium (Cd) are often detected in livestock slurry. These metals originating from feed additives and veterinary treatments can accumulate in the soil, especially in the topsoil layer, with their repeated application. Elevated metal concentrations can be toxic to soil microorganisms and plants, inhibiting enzymatic activities, and can potentially enter the food chain through forage crops. Zinc (Zn) and copper (Cu) have been shown to disrupt microbial diversity and function when their concentrations exceed certain thresholds [87]. Slurry contains organic matter that can contribute to soil C stocks, improving soil structure and water retention. However, the decomposition of organic matter in slurry also releases carbon dioxide and methane, which are potent greenhouse gases. With proper slurry management, their incorporation into the soil can even enhance carbon sequestration while minimizing emissions. Livestock slurry boosts soil fertility, but requires careful management to prevent acidification, metal buildup, and emissions.

### 5.3. Air Pollution

The application of livestock slurry to agricultural fields contributes significantly to air pollution through the emission of various gases, including ammonia, methane, nitrous oxide, and hydrogen sulfide [88,89,90,91].

Methane is a potent greenhouse gas with a global warming potential significantly higher than carbon dioxide over a 20-year period. This gas is mainly produced during the anaerobic decomposition of organic matter in slurry, which is then released into the atmosphere during and after field application. Mitigation strategies, such as incorporating slurry into the soil, can reduce methane emissions by limiting the exposure of slurry to anaerobic conditions. To reduce methane emissions in livestock production, it is necessary to identify all the factors associated with its generation. In a study by Hilgert et al. [89], the influence of storage temperature and chemical composition on methane emissions from pig and dairy manure was assessed. After 90 days of incubation at temperatures ranging from 5 °C to 25 °C, the authors reported that maximum methane emissions were observed at higher temperatures, such as 20 °C and 25 °C for pig manure and 25 °C for dairy slurry. However, the levels of volatile fatty acids (VFA) during storage revealed a potential inhibition of methane production at higher temperatures where levels of VFA were higher, challenging the hypothesis of higher temperatures being one of the main factors for higher methane emissions.

The application of livestock slurry to soils introduces nitrogen, which can undergo nitrification and denitrification processes, leading to the production and emission of nitrous oxide. Nitrous oxide is a potent greenhouse gas with global warming potential. The emission of nitrous oxide is influenced by type of treatment, storage duration, and carbon content of manure. In general, the ratio of nitrous oxide to N increases with higher acidity levels, nitrate concentration, and reduced moisture [88,92]. Factors influencing nitrous oxide emissions include soil type, temperature, moisture content, and the timing and method of slurry application. For instance, the use of chemical amendments like alum and iron chloride in slurry has been shown to increase nitrous oxide emissions significantly [88]. Other authors assessed the effects of application techniques and N-stabilizing strategies to reduce nitrous oxide emission from grasslands. Nyameasem et al. [93] estimated the effects of different fertilizers applied as calcium ammonium nitrate, and untreated or treated cattle slurry on nitrous oxide emissions. Slurry pH was adjusted to six with sulfuric acid and applied using trailing shoes or treated with 3,4-dimethyl pyrazole phosphate and applied via slot injection. The study reported inconsistent effects on soil mineral N content, cumulative nitrous oxide emission, and N yield. Cumulative emissions of nitrous oxide ranged from 0.1 to 2.9 kg N ha^−1^ year^−1^, and a positive relationship between slurry value and crop biomass yield was observed. This field experiment also highlighted the variations in nitrous oxide by soil and climatic factors.

Ammonia present in the slurry can volatize as ammonia gas, especially under warm, moist, and alkaline conditions. Particle size is also a concern and is linked to respiratory issues. Every year, it is estimated that 15% of the N in animal manure is lost to the atmosphere as ammonia [21]. In a study to determine how the chemical amendment of slurry affects losses of ammonia, methane, nitrous oxide, and carbon dioxide, Brennan et al. [88] reported a reduction in ammonium emissions by 92, 54, 65, and 77% using alum, iron chloride, polyaluminum chloride, and biochar, respectively, when compared to the slurry control. In a study assessing the impacts to greenhouse gas and ammonia emissions in pilot-scale slurry storage, Misselbrook et al. [91] tested different temperatures (8, 11, and 17 °C) and mitigation practices of slurry acidification and covering with granulated clay. Both methane and ammonia gases were influenced by temperature over the storage period, reduced with slurry acidification, and covering with clay granules effectively reduced ammonia emissions.

Hydrogen sulfide is produced during the anaerobic decomposition of sulfur-containing organic compounds in slurry. It is a toxic gas with a characteristic rotten egg odor and poses health risks to human and animals at elevated concentrations. Emissions of hydrogen sulfide are particularly concerning during slurry agitation and application, necessitating proper management practices to minimize exposure. In a study to assess the emission and dispersion of hydrogen sulfide gas during slurry agitation in typical configurations of indoor and outdoor storage, with and without slatted flooring, Gyte and Kelsey [90] reported that hydrogen sulfide gas levels increased rapidly following agitation and remained generally high until agitation ceased. The highest gas concentrations were observed in indoor storage facilities with slatted floors. To reduce the risks and exposure to hydrogen sulfide in farms, the development of new alternatives to store and handle slurry is necessary.

### 5.4. Impact on Biodiversity

Under natural conditions, nutrients are integrated into the soil through biological activities originating from a complex network of organisms below and above the soil, such as bacteria, fungi, protozoa, micro and macrofauna, algae, and plants. The chain of interactions between these organisms maintains a cycle of consumption and replacement of these nutrients in a balanced way, as detailed in Section 4, maintaining soil quality [92]. However, it is common to associate soil quality with a high availability of nutrients (N, K, P, C) in the soil to meet the metabolic demand of plants, whether from crops or pastures. To quickly supply these components to the soil, the practice of fertilization with mineral or organic fertilizers is adopted.

Cattle slurry, due to its richness in nutrients, acts as an organic fertilizer, nourishing the soil biota. Under natural conditions, it is estimated that in 1 g of soil, there are approximately 10^9^ bacterial cells of different taxa [94]. When not pasteurized, slurry also carries microorganisms present in the intestinal microbiota of cattle, which can affect soil biodiversity. Thorn et al. [95] reported a significant change in the bacterial community after slurry management, but this change began to regress after the third day of slurry treatment. In these first days, the soil respiration rate is high due to the abundance of microbes and the high carbon content in the slurry. After this period, the resources added by fertilizer begin to decay and the microbes that are not able to live in the soil environment tend to die, giving way to the natural soil microbiota. Among the most abundant phyla after slurry management in soil are *Bacteroidota*, which has species that secrete a set of carbohydrate-active enzymes and are considered soil quality indicator species [96], and the *Bacillota* phylum, composed of bacteria that carry the gene related to the denitrification process [97]. However, in the same paper, Thorn et al. [95] also reported high nitrification rates up to 90 days after soil management of slurry. As already seen, the effects of long-term slurry application can result in changes, among other things, in soil acidity, soil nitrification, and an increased solubility of toxic elements, events that can affect plants’ development [48].

Mycorrhiza is a structure formed by the mutualistic union between fungi and plant roots. It increases the area of nutrient absorption for the plant while providing a source of carbohydrates for the fungi. Microhabitats are maintained amidst the mycorrhizal webs, and are home to specific microorganisms that participate in nutrient cycling. Cai et al. [98] reported a decrease in the richness of these microorganisms after slurry management, probably due to the disturbance of these microhabitats [98].

The homogenization of the landscape in the formation of pasture for livestock and the use of fertilizers reduces the food resources of the local flora and fauna. The formation of pastures generally requires the introduction of specific grasses for livestock, and when supplemented with manure, they increase biomass and generate a decrease in local diversity [99]. The impact of slurry on local flora is still debated. When analyzing the diversity of plants in pastures with different types of management, Gros et al. [100] noted a decrease in the number of botanical species in pastures treated with slurry compared to pastures without fertilizers, while Pornaro et al. [101] also reported a decrease in phytosociological classes; that is, a reduction in ecologically correlated species in areas with the application of slurry. The decline in botanical diversity also reduces fauna diversity, particularly of invertebrates responsible for pollination [102].

Threatened species are, for the most part, restricted to locations with specific resources and conditions for their survival and reproduction. When comparing diversity in different pastures, Gros et al. [100] did not identify a considerable difference in the number of carabid species present in pastures treated with slurry and pastures without fertilizers; however, she reported the presence of three vulnerable carabid species only in pastures without any treatment. In other words, by homogenizing the landscape, physically and chemically, the particularities of its native composition are lost, restricting the presence of specialist and vulnerable species, while contributing to the increase in generalist species that, although beneficial, can become pests. The effect of slurry management on biological diversity and its interactions is, in fact, debated, but there is a need to deepen studies on the factor of time, and how successful slurry management affects biodiversity in the long term [103].

### 5.5. Antimicrobial Resistance

The presence of antibiotics, antibiotic-resistant bacteria (ARB), and antibiotic resistance genes (ARG) in livestock slurry raises concerns about their persistence in soil, potential uptake by crops, and dissemination into water systems, ultimately threatening public and environmental health [94]. Livestock slurry often contains residues of antibiotics administered to animals for therapeutic, prophylactic, and growth-promoting purposes. Common antibiotics detected in slurry include tetracyclines, sulfonamides, macrolides, and fluoroquinolones. Antibiotics can persist in slurry for extended periods, depending on environmental conditions and the physicochemical properties of the compounds. ARG from soil bacteria can be transferred to human pathogens through indirect pathways, such as irrigation water, food crops, and dust particles. This fact questions the role of agricultural environments in the global AMR crisis [99]. Soil microbial communities might be impacted by the presence of antibiotic residues and resistant bacteria, even though significant effects are not always observed at environmental concentrations. Past studies have demonstrated that antibiotics tend to accumulate in surface soil layers. As a result, the mobility of these antibiotics through the deeper layers of the soil profile is significantly restricted [100].

The presence of antibiotic resistance genes in soil and runoff is influenced by the application of slurry, with certain genes showing significant reductions in runoff when setback distances are increased [102]. In a study assessing the impact of residual antibiotics on the microbial decomposition of livestock manure, Fang et al. [96] reported that antibiotic exposure hindered the decomposition rate and nutrient release, regardless of the antibiotic and livestock manure used in the experiment. This might affect soil fertility, carbon sequestration, and potentially lead to nutrient leaching. The lower decomposition rate could be explained by the shift in microbial diversity and abundance, activity of digestive enzymes, and co-occurrence patterns.

Shawver et al. [104] studied the long-term impact of repeated manure additions from cattle under different antibiotic treatments on microbial communities and ARG abundance. The three-year study revealed that manure changed the soil bacterial and fungal communities, while manure from cattle under antibiotic treatment altered only soil bacteria, but both led to persistent effects, suggesting that impacts to soil microbial communities can persist for long periods. Pig slurry often contains antibiotic-resistant bacteria, including multi-resistant strains of *E. coli* and *Salmonella*, which pose a risk of spreading resistance genes in the environment [105]. In 2020, Rasschaert et al. [105] analyzed 89 pig slurry samples. From these, 51 samples harbored *Salmonella*, and 65% of *Salmonella* isolates were resistant to five antibiotics, 52 samples contained *E. coli* isolates resistant to ciprofloxacin and 22 resistant to cefotaxime. All isolates resistant to ciprofloxacin and cefotaxime were multi-resistant, with resistance up to nine and eight antibiotics, respectively.

Despite the problems caused by pathogens and multi-resistant bacteria, microorganisms play a crucial role in mitigating AMR from livestock slurry through various mechanisms. Certain bacteria, such as those in the genera *Pseudomonas*, *Bacillus*, and *Achromobacter*, can degrade antibiotic residues, reducing their persistence in the environment [103]. Additionally, microbial communities in soil and manure can outcompete ARB, limiting their proliferation and horizontal gene transfer [97]. Livestock slurry contributes to the spread of antibiotic resistance, but soil microbes can help mitigate its impact through biodegradation and competition.

## 6. Regulations for Slurry Application in Ireland and Europe, and Environmental Goals

Ireland’s approach to managing slurry application is heavily influenced by the European Union’s Nitrates Directive (91/676/EEC) [15]. This directive aims to protect water quality by preventing nitrates from agricultural sources from polluting groundwater and surface water.

Under this framework, Ireland developed its own Nitrates Action Program (NAP), which outlines strict guidelines on the storage, timing, and application of slurry to minimize nutrient runoff and environmental degradation. The regulations specify closed periods during which slurry application is prohibited, ensuring that nutrients are not applied when they are more likely to leach into water bodies due to rainfall or saturated soil conditions. Ireland’s roadmap for tackling climate change is the Climate Action Plan. Its goal is to guide the country toward achieving a national climate target: a climate-resilient, biodiversity-rich, and environmentally sustainable economy that reaches net-zero emissions by 2050 [16].

The regulations governing slurry application in Ireland are essential for ensuring the sustainable use of livestock slurry as a fertilizer. By enforcing restrictions on storage and application timing, they mitigate the risks of nutrient runoff and water contamination, aligning with environmental objectives. Additionally, Ireland’s Climate Action Plan underscores the country’s commitment to a sustainable agricultural sector, and through livestock slurry management, protecting biodiversity and reducing methane emissions. These regulations play a role in optimizing slurry application while minimizing adverse environmental impacts. As discussed throughout this paper, microorganisms are essential to nutrient cycling, C sequestration, and emissions reduction, therefore being a natural solution to enhance slurry management efficiency. The adoption of microbial strategies could significantly improve soil health, nutrient availability, and methane reduction, complementing Ireland’s sustainability goals.

### 6.1. Policies Promoting Sustainable Slurry Management Practices

To encourage sustainable agricultural practices, Ireland has implemented several policies aimed at reducing the environmental impact of slurry application. The Good Agricultural Practice (GAP) regulations form a cornerstone of Ireland’s strategy, emphasizing practices that limit nutrient losses [106]. These regulations advocate for precision in nutrient management through soil testing and nutrient management planning to ensure that slurry application rates match crop needs.

The Agricultural Sustainability Support and Advisory Program (ASSAP) is another initiative designed to support farmers in adopting sustainable slurry management practices. This collaborative program between governmental bodies and agricultural organizations provides advisory services that promote the adoption of best management practices (BMPs) to enhance water quality. The farmer and advisor will agree on where to focus improvements or actions on the farm, with efforts such as improved nutrient management with more targeted use of slurry and fertilizer, implementing new approaches to land management to reduce nutrient losses in critical source areas, and better general farmyard management and practices [107,108].

The Common Agricultural Policy (CAP) aims to promote sustainable agricultural practices, with the aim to reduce ammonia emissions from livestock farming. It supports farmers in implementing techniques that improve air quality and align with the EU’s environmental demands [109].

In Spain, the Royal Decree 1051/2022 established laws for sustainable nutrition in agricultural soils. It bans the use of plate, fan, or cannon systems for moist organic materials, and mandates manure burial within 24 h to reduce emissions. Solid manure piles may only remain in fields for 10–20 days, depending on the treatment performed. The decree promotes low-emission fertilizers, imposes stricter controls on urea use if national levels exceed 30%, and regulates nitrogen application timing. Organic liquid waste must be applied at least two months before harvest, with some exceptions. Farmers must maintain records of nutrient inputs, and waste managers must provide environmental identification numbers for traceability [110].

In Portugal, the ordinance no. 79/2022 defines management laws for livestock effluents, under the New Livestock Activity Regime (NREAP) from Decree–Law no. 81/2013. Only composting facilities processing up to 27,375 tons per year are exempt from licensing, provided that they meet technical and environmental requirements. Composting slurry must adhere to strict temperature, humidity, and odor standards to prevent contamination. Furthermore, when handling animal by-products, facilities must obtain a veterinary control number (NCV) from the General Directorate of Food and Veterinary Affairs (DGAV) [111].

### 6.2. Incentives and Support Programs for Farmers to Adopt Best Practices

The Irish government has established various incentives and support programs to assist farmers in implementing best practices for slurry management. The Green, Low-Carbon, Agri-Environment Scheme (GLAS) [112] offers financial support to farmers who adopt environmentally friendly practices, such as low-emission slurry spreading (LESS) techniques. These techniques, including trailing shoe and dribble bar systems, help reduce ammonia emissions and improve nutrient uptake by crops.

Additionally, grants are available under the Targeted Agricultural Modernization Scheme (TAMS) for farmers looking to invest in modern equipment that supports sustainable slurry management, such as slurry storage tanks and LESS application machinery. These programs not only help reduce the environmental footprint of farming, but also align with Ireland’s broader goals of meeting climate action targets [113].

### 6.3. Best Management Practices to Mitigate Environmental Impacts

Implementing best management practices (BMPs) is essential for minimizing the environmental impacts associated with slurry application. Key BMPs include [114]:

Timing and Zone of Application: Slurry should be applied during periods of active crop growth when nutrient uptake is highest. The timing should avoid wet weather and saturated soil conditions to prevent runoff and leaching. For the first two weeks after the spreading period begins, slurry must be kept at least 10 m away from rivers, streams, watercourses, and drains. After this period, the required distance reverts to 5 m. Table 6 illustrates safe buffer distances from water bodies for spreading organic fertilizers.

Application Rates: Matching the rate of application of organic fertilizers with the growth rates of grass or crops is crucial, especially during early spring when growth is minimal. Overapplying slurry beyond the crop’s nutrient demand can result in nutrient leaching. Therefore, lower application rates of organic fertilizers are advised during this period. By following these BMPs and adhering to regulatory requirements, Irish farmers can play a significant role in safeguarding water quality, reducing greenhouse gas emissions, and contributing to the overall sustainability of the agricultural sector.

In the west of France, in Bretagne, collective slurry management strategies have been evaluated for their environmental performance. Scenarios include the transfer of slurry to cropland and its treatment in a biological station. The transfer, which involves injecting slurry into cropland, has been found to be environmentally safer when compared to treatment. There is lower acidification, eutrophication, and non-renewable energy use, as the energy saved by reducing mineral fertilizer use compensates for the energy required for transport [115].

Researchers studied different ways to manage manure in Sweden, Denmark, France, and Italy, including changes in storage, separating solid and liquid parts, and burning the solid part. Findings showed that manure handling can greatly impact greenhouse gas emissions and C storage. The effect of new technologies depends on how farms operate and the local climate. The study showed that greenhouse gas estimates can be inaccurate if manure management practices or climate conditions are not properly considered for a specific country or region. The effectiveness of different manure management methods depends on the existing farming practices and climate [116]. Proper slurry management also contributes to the European Green Deal Focus of achieving no net emissions of greenhouse gas by 2050 [117].

### 6.4. The Agriculture and Food Development Authority (Teagasc)

In Ireland, Teagasc (the Agriculture and Food Development Authority) is the primary agency responsible for supporting farmers. It provides a comprehensive system of research, advisory services, and education, ensuring that Irish agriculture remains sustainable, competitive, and resilient. Teagasc supports farmers by conducting agricultural research to improve productivity and sustainability and developing new farming technologies. Teagasc also supports climate action and environmentally friendly farming [118]. They also provide expert advice on farm management, business planning, and efficiency, help farmers access government schemes, grants, and financial support, and ensure compliance with national and EU regulations [119].

Teagasc supports farming through education with agricultural colleges and training programs for farmers, offering courses on modern farming, sustainability, and agribusiness [120]. Countries can benefit from adopting a similar approach that combines research, advisory services, and education to create a more sustainable and reliable agricultural industry. By supporting farmers, governments can ensure food security, economic stability, and environmental protection while helping their agricultural sectors face global challenges.

### 6.5. Considerations of Livestock Slurry Application to Irish Soil and Environment

Ireland’s unique soil and environmental conditions influence the application of livestock slurry on fields. Emissions and environmental impact are influenced since livestock slurry use is a major source of ammonia and greenhouse gas emissions in Ireland. Different techniques, such as the trailing shoe method, can reduce ammonia volatilization, but can also increase direct nitrous oxide emissions. Timing is also crucial, since spring applications are more effective in reducing emissions compared to summer applications because of soil and climatic conditions that enhance crop growth [121,122].

Soil and crop management need to be considered since the method of slurry application affects nutrient uptake and emissions. Injection methods may reduce ammonia emissions significantly without increasing nitrous oxide emissions, maintaining crop yields [123,124]. The acidification of slurry before application can also reduce ammonia emissions and increase N availability for crops [125]. Slurry application can lead to nutrient and metal runoff, especially with post-application rain. This runoff is more pronounced with dairy cattle slurry compared to treated biosolids [126]. Proper management and timing can mitigate these losses and improve soil quality by increasing organic C and nutrient availability [121]. Low emission spreading methods, such as trailing shoe and shallow injection, are indicated to increase the number of suitable days for slurry application in spring, especially on well-drained soils. These methods help in managing soil moisture, crucial for effective slurry application [127].

### 6.6. Comparable Institutions to Teagasc in Europe

In Portugal, the APA (Agência Portuguesa do Ambiente), or the Portuguese Environment Agency, is the national authority responsible for environmental policies, ensuring sustainable resource management and regulating activities connected to waste management, water resources, air quality, climate change, and biodiversity protection. It operates under the Ministry of Environment and Climate Action, enforcing environmental legislation, issuing permits, conducting inspections, and promoting sustainability initiatives. It also plays a crucial role in waste regulation, enforcing the general waste management regime (RGGR) and ensuring the proper handling of slurry, fertilizers, and organic waste [128].

In the UK, FWAG (Farming and Wildlife Advisory Group), a non-profit organization, provides environmental advice to farmers, helping them adopt sustainable practices in land management and comply with environmental regulations. It supports farmers with agri-environment schemes, soil and water management, habitat conservation, and reducing agricultural pollution. It also offers training and farm conservation [129].

The Environment Agency (EA) is a government regulatory body in England, and it is responsible for protecting the environment. It enforces laws related to water quality, waste management, flood protection, air pollution, and land use, including regulations that affect agriculture. The EA ensures that English farmers comply with slurry storage rules, nitrate vulnerable zones, pesticide regulations, and pollution control laws to prevent damage to rivers, soil, and air. The EA also provides permits for water abstraction, waste disposal, and intensive farming operations, ensuring sustainable land and resource management [130].

Chambers of Agriculture are regional advisories present in France and Austria, providing practical farming advice, research-based innovations, and compliance support for agricultural regulations. They connect farmers and the government, implementing EU agricultural policies (CAP), environmental regulations, and sustainability initiatives [131].

In France, the French Ministry of Agriculture and Food Sovereignty is responsible for regulating and shaping national agricultural policies in compliance with EU CAP rules. It plays a crucial role in sustainable farming practices, promoting agroecology, organic farming, and precision agriculture. The Ministry enforces the Nitrates Directive [132].

In Germany, the Federal Ministry of Food and Agriculture (BMEL) sets policies for sustainable farming, nutrient management, and livestock production, while enforcement is handled at a state level. German agriculture is marked by precision farming technologies, organic certification schemes, and climate-adaptive soil conservation techniques, all promoted by BMEL research and funding programs [133].

Austria’s Federal Ministry of Agriculture, Regions, and Tourism is a strong influence in sustainable and organic farming. It also implements EU CAP regulations, promoting integrated nutrient management and environmentally friendly livestock practices via eco-schemes and funding. Slurry management is controlled under the Austrian Nitrate Action Program, which mandates long-term manure storage, with strict closed periods for slurry spreading and precision application methods to minimize nitrogen losses [134].

## 7. Future Directions and Research Needs

The management of livestock slurry is essential for maintaining soil fertility, reducing environmental risks, and enhancing pasture productivity. This study underscores the critical role of soil microorganisms in regulating nutrient dynamics, particularly in the cycling of N, P, C, and S, which are fundamental for plant nutrient intake and overall soil fertility. Through microbially driven pathways such as nitrification, denitrification, and phosphate solubilization, microorganisms regulate nutrient availability and organic matter decomposition. A balanced interplay of these processes is essential for maintaining grassland productivity, stability, and biodiversity.

The application of organic materials, including cattle manure, has been shown to improve microbial communities and enhance productivity in the livestock industry. Slurry increases soil C inputs, stimulating microbial biomass and shaping microbial community structures. However, if mismanaged, livestock slurry can contribute to soil degradation, nutrient leaching, greenhouse gas emissions and the spread of antimicrobial resistant genes.

Regulatory frameworks such as the Nitrates Directive and Ireland’s Nitrates Action Program (NAP), along with initiatives like LESS, GLAS, and TAMS, have been instated in Ireland and across Europe. Nonetheless, gaps remain in their practical adoption and integration in farming practices. This study highlights key areas of improvement, emphasizing the need for greater promotion of agricultural sustainability and broader dissemination of information on microbial interventions as a tool for more efficient slurry management.

Microbial-based strategies present a promising complement to current regulations. For instance, methanotrophic bacteria and slurry acidification can significantly reduce methane emissions, while phosphate-solubilizing bacteria can improve P bioavailability. Integrating these tactics into existing frameworks could further minimize environmental impacts and optimize nutrient assimilation, ultimately contributing to a more productive and sustainable livestock system.

The development of microbial solutions tailored for nutrient decomposition and emission reduction is a vital promise for sustainable agriculture. Future studies must prioritize testing microbial strategies across a range of soil types and climatic conditions to ensure their efficacy. This should improve nutrient cycling and also minimize the environmental footprint of slurry-fertilized pastures.

The integration of advanced technologies such as sensors, drones, and artificial intelligence into slurry management practices represents an inevitable adaptation. These tools offer real-time monitoring and optimization of slurry application, enabling precision agriculture that enhances nutrient use efficiency while reducing greenhouse gas emissions and leaching.

Future research should also emphasize long-term studies focusing on soil health, biodiversity, and C sequestration. This will provide data to assess the viability of sustainable practices, promoting greater acceptance among farmers. Understanding these long-term impacts is essential for building agricultural systems that align with Ireland’s environmental goals.

Finally, the role of policy and incentives cannot be overstated. Establishing frameworks that support the adoption of sustainable practices is essential. Government and policymakers should consider subsidies, grants, and technical assistance programs to encourage farmers to transition to adopt environmentally friendly methods. By aligning economic incentives with environmental objectives, these measures can drive the widespread implementation of innovative practices and contribute to achieving national sustainability targets. Mitigation measures such as proper slurry treatment through composting or anaerobic digestion, timing applications to avoid rain, and using injection methods instead of surface spreading can help minimize these risks.

## 8. Conclusions

### 8.1. The Role of Livestock Slurry in Sustainable Agriculture

This study confirms that livestock slurry is a valuable organic fertilizer that enhances soil fertility, supports microbial activity, and improves pasture productivity. Its application must be carefully managed to mitigate environmental risks, including nutrient runoff, water contamination, greenhouse gas emissions, and the spread of antibiotic resistance. Sustainable slurry management facilitates optimal nutrient utilization while minimizing environmental impacts.

### 8.2. Microbial Contributions to Nutrient Cycling and Environmental Sustainability

Microorganisms are shown to be key players in nutrient cycling and organic matter decomposition. These processes increase the availability of essential nutrients to plants, improve soil structure, and potentially mitigate antimicrobial resistance through natural degradation mechanisms. Microbial strategies offer promising solutions for reducing environmental emissions and enhancing crop productivity.

### 8.3. Environmental and Regulatory Approaches in Slurry Management

Regulatory frameworks governing slurry management and sustainable slurry management practices, such as precision application methods and microbial-based strategies, are essential to maximize its benefits while minimizing ecological harm. This study identifies gaps in integration with emerging microbial-based solutions. By integrating scientific advancements with responsible farming practices, livestock slurry can continue to be a key component of sustainable agriculture while reducing its environmental footprint.

## Figures and Tables

**Table 1:** Types of microorganisms involved in nutrient cycling and their specific roles.

Micro-Organism	Key Groups	Specific Roles	Examples	References
Bacteria	Decomposers (heterotrophic bacteria)	Breaks down organic matter into simpler inorganic compounds like CO_2_, NH_4_^+^, and phosphates	*Bacillus*, *Pseudomonas*, *Clostridium*	[55]
	Nitrogen-cycling bacteria	Nitrogen fixers convert atmospheric nitrogen (N_2_) into ammonia (NH_3_), which can be used by plants	Symbiotic nitrogen-fixers (*Rhizobium*, *Frankia*)	[6,56]
			Free-living nitrogen-fixers (*Azobacter*, *Cyanobacteria*, e.g., *Anabaena*, *Nostoc*)	
		Nitrifying bacteria converts NH_3_ into nitrites (NO_2_^−^) and then into nitrates (NO_3_^−^) which can be absorbed by plants	Ammonia-oxidizing bacteria (*Nitrosomonas*),	[6,56]
			nitrite-oxidizing bacteria (*Nitrobacter*)	
		Denitrifying bacteria converts NO_3_^−^ back into N_2_ and releasing it into the atmosphere	*Pseudomonas*, *Paracoccus*, *Clostridium*	[6,56]
	Phosphate-solubilizing bacteria	Converts insoluble phosphates in the soil into soluble forms, making phosphorous available to plants	*Pseudomonas*, *Bacillus*, *Rhizobium*	[49,57]
	Sulfur-cycling bacteria	Sulfur-oxidizing bacteria converts hydrogen sulfide (H_2_S) into sulfate (SO_4_^2−^) which is then usable by plants	*Thiobacillus*, *Beggiatoa*	[6,49]
		Sulfur-reducing bacteria reduces SO_4_^2−^ back to H_2_S in anaerobic conditions	*Desulfovibrio*, *Desulfotomaculum*	[49,58]
Fungi	Saprotrophic fungi	Decompose organic matter, releasing nutrients back into the soil	*Aspergillus*, *Penicillium*, and various *basidiomycetes* (wood-decaying fungi)	[6,56]
	Mycorrhizal fungi	Forms symbiotic relationship with plant roots, enhancing nutrient uptake (especially phosphorous) while receiving carbohydrates from the plant	Ectomycorrhizal fungi (*Boletus*, *Amanita*),	[4,5,59]
			arbuscular mycorrhizal fungi (AMF) (*Glomus*, *Rhizophagus*)	
	Endophytic fungi	Live within plant tissues and assist in nutrient uptake and protection from environmental stresses	*Neotyphodium*, *Fusarium* (some species)	[49,56]
Archaea	Methanogenic archaea	Produces methane (CH_4_) as a byproduct of anaerobic decomposition of organic matter	*Methanobacterium*, *methanosarcina*	[6]
	Ammonia-oxidizing archaea	Converts NH_3_ to NO_2_^−^ in the N cycle, particularly in marine and soil environments	*Nitrosopumilus*, *Nitrososphaera*	[56]
Protozoa		Consumes bacteria to release N_2_ and P in forms that are more readily available for plant uptake	Amoebas, ciliates, flagellates	[6,49]
Algae and cyanobacteria		Photosynthetic carbon fixation, they convert CO_2_ into organic matter forming the base of the food web in aquatic environments	*Chlorella* (algae), *Spirulina* (cyanobacteria)	[49]
		Nitrogen fixation, some cyanobacteria fix atmospheric N and enrich ecosystems with bioavailable N	*Anabaena*, *Nostoc*	[56,60]

**Table 2:** The roles of key microbes involved in the carbon cycle and their impact on the plants.

Microbial Activity	Key Microorganisms	Mechanism of Microbial Activity	Impact on Plants	References
Fixation	Cyanobacteria (*Anabaena*, *Spirulina*, *Nostoc*), algae, chemoautotrophic bacteria (*Nitrobacter*, *Thiobacillus*)	Photosynthetic microorganisms use sunlight to convert CO_2_ into carbohydrates via photosynthesis	Contributes organic carbon to soil and aquatic ecosystems, enriching carbon pools that plants rely on indirectly	[5,49]
Decomposition	Saprotrophic fungi (*Penicillium*, *Aspergillus*, basidiomycetes), bacteria (*Bacillus*, *Pseudomonas*), Actinomycetes (*Streptomyces*)	Decomposes microorganisms, degrades complex organic matter into simpler compounds like CO_2_ and methane (CH_4_). Enzymes like cellulase, ligninases, and amylases break down polysaccharides, lignin, and other organic compounds	Recycles organic carbon into the soil, enriching it with nutrients and carbon compounds that plants can utilize for growth	[5,56,61]
Humus formation	Fungi and actinomycetes	Microbial activity converts decomposed organic material into humus, a stable form of organic carbon in the soil. Soil bacteria and fungi aggregate organic matter with soil particles, stabilizing carbon in the soil matrix	Enhances soil structure and fertility, improving water retention and nutrient availability for plant roots.	[5,7,56]
Symbiotic associations	Mycorrhizal fungi (*Glomus*, *Amanita*), nitrogen-fixing bacteria (*Rhizobium*)	Fungi mutualistic associations with plant roots aiding in nutrient acquisition and use carbon exuded by roots for their growth and metabolism. Nitrogen-fixing bacteria converts atmospheric nitrogen into usable forms for plants in exchange for carbon compounds produced by the plant through photosynthesis	Supports plant growth by improving nutrient uptake efficiency, which is dependent on microbial carbon metabolism	[6,56,62]
Methane production and oxidation	Methanogenic archaea (*Methanobacterium*, *Methanosarcina*), methanotrophic bacteria (*Methylococcus*, *Methylobacter*)	Methanogens (anaerobic archae) produce CH_4_ by breaking down organic compounds in oxygen-deprived environments (e.g., rice paddies). Methanotrophs (aerobic bacteria) oxidize CH_4_ into CO_2_, reducing its release into the atmosphere	Maintains carbon balance in wetland and flooded ecosystems where methane could otherwise accumulate	[5,48,49]
Sequestration	Arbuscular mycorrhizal fungi (AMF) (*Glomus*, *Rhizophagus*), soil bacteria (*Actinobacteria*, *Bacillus*)	Soil microbes process C inputs (e.g., plant residues, root exudates) into stable organic compounds or aggregates them with minerals. AMF plays a significant role in forming soil aggregates that traps carbon.	Increases soil fertility and carbon storage, creating a sustainable growth environment for plants	[5,56,63]
Rhizosphere cycling	Rhizobacteria (*Pseudomonas*, *Azospirillum*), fungi (*Aspergillus*, *Trichoderma*)	Plants release root exudates (sugars, amino acids) into the rhizosphere, which serve as carbon sources for microbial growth. Microbial activity mineralizes these compounds, releasing CO_2_ or converting them into stable organic forms	Promotes nutrient cycling and protects roots from pathogens through beneficial microbial interactions	[7,56,63]
Respiration	Heterotrophic bacteria (*Bacillus*, *Pseudomonas*) saprophytic fungi (*Penicillium*, *Aspergillus*)	Soil microbes metabolize organic matter or root exudates, releasing CO_2_ as a byproduct of energy generation. This CO_2_ can be absorbed by plants for photosynthesis	Sustains the atmospheric carbon pool necessary for photosynthesis	[49,54,64]

**Table 3:** The roles of key microbes involved in the nitrogen cycle and their impact on plants.

Microbial Activity	Key Microorganisms	Mechanism of Microbial Activity	Impact on Plants	References
Fixation	Symbiotic bacteria (*Rhizobium*, *Bradyrhizobium*), free-living bacteria in anaerobic conditions (*Azobacter*, *Clostridium*), associative bacteria (*Azospirillum*)	Nitrogen-fixing bacteria and archaea use an enzyme called nitrogenase to break the strong triple bond in N_2_ gas and convert it into NH_3_ Symbiotic nitrogen fixation: bacteria like Rhizobium form a symbiotic relationship with the roots of leguminous plants. These bacteria live in root nodules and provide the plant with N in exchange for carbohydrates Free-living N fixers: bacteria like *Azotobacter* and *Clostridium* fix nitrogen independently in the soil Cyanobacteria: these are photosynthetic bacteria found in aquatic environments that can also fix N	Converts atmospheric nitrogen (N_2_) gas into ammonia (NH_3_) that can be used by plants	[5,60,67,68]
Nitrification	Ammonia-oxidizing bacteria (*Nitrosomonas*, *Nitrosospira*), nitrite-oxidizing bacteria (*Nitrobacter*, *Nitrococcus*)	Step 1: ammonia oxidation NH_3_ is oxidized to NO_2_^−^ by bacteria (such as *Nitrosomonas* or *Nitrosococcus*) 2NH_3_ + 3O_2_ → 2NO_2_^−^ + 2H^+^ + 2H_2_O Step 2: nitrite oxidation NO_2_^−^ is further oxidized to NO_3_^−^ by bacteria (such as *Nitrobacter* or *Nitrospira*) 2NO_2_^−^ + O_2_ → 2NO_3_^−^	Converts N into water-soluble forms (NO_3_^−^) that can be easily transported through the plant’s vascular system	[5,56,69]
Ammonification	Decomposer bacteria (*Bacillus*, *Pseudomonas*, *Clostridium*), fungi (*Aspergillus*, *Penicillum*)	Microorganisms decompose proteins, nuclei acids, and other nitrogenous compounds, releasing NH_3_ or NH_4_^+^ into the soil	Serves as a direct N source for plants, especially in acidic soils where NH_4_^+^ remains stable	[56,65]
Denitrification	Facultative anaerobic bacteria (*Pseudomonas*, *Paracoccus*, *Clostridium*)	Denitrifying bacteria use NO_3_^−^ as an alternative to oxygen for respiration. This leads to the reduction of NO_3_^−^ to N_2_ gas Step 1: NO_3_^−^ is reduced to NO_2_^−^ Step 2: NO_2_^−^ is further reduced to nitric oxide (NO), N_2_O and finally N_2_ gas	Reduces N availability for plants and avoids excessive denitrification, leading to N loss from soils	[6,49,56]
Regulation in anaerobic conditions	Bacteria (*Brocadia*, *Kuenenia*)	Converts ammonium (NH_4_^+^) into nitrogen gas (N_2_) under anaerobic conditions	Reduces N availability in anaerobic soils, helps regulate N levels and prevent harmful N compound build-up	[4,56]

**Table 4:** The roles of key microbes involved in the phosphorus cycle and their impact on plants.

Microbial Activity	Key Microorganisms	Mechanism of Microbial Activity	Impact on Plants	References
Solubilization	Bacteria (*Pseudomonas*, *Bacillus*, *Rhizobium*, *Enterobacter*), fungi (*Aspergillus*, *Penicillium*)	Microorganism releases organic acids (e.g., citric acid, gluconic acid) that lower the soil pH, dissolving insoluble phosphate compounds. Enzymes like phosphatases break down organic P compounds.	Converts insoluble phosphorus into soluble forms (H_2_PO_4_^−^ and HPO_4_^2−^) that can be absorbed by plants	[5,56,57]
Mineralization	Bacteria (*Pseudomonas*, *Bacillus*), fungi (*Aspergillus*, *Penicillium*)	Microbial decomposition of dead plant and animal material releases organic phosphorus. Enzymes like acid phosphatases, alkaline phosphatases, and phytases help hydrolyze organic P compounds into inorganic phosphate	Releases inorganic forms (orthophosphate) that plants can use	[5,71]
Mycorrhizal associations	Arbuscular mycorrhizal fungi (AMF) (*Glomus*, *Rhizophagus*), ectomycorrhizal fungi (*Amanita*, *Boletus*)	The fungi absorbs and transfer P from soil to the plant	Enhances the plant’s phosphorous uptake	[56,70]
Organic P cycling	Bacteria (*Bacillus*, *Pseudomonas*), fungi (*Aspergillus*, *Penicillium*)	Microorganism-producing enzymes like phytases and phosphoesterases hydrolyze organic P compounds	Degradation of phytates and enhances plant’s P uptake	[51,56]
Immobilization	Soil bacteria and fungi	P is released back into the soil upon microbial death and decomposition	Stores P in microbial biomass, temporarily reducing its availability in the soil	[49,62]
Biofilm formation and transport	Mycorrhizal fungi hyphae and rhizosphere microorganisms	Microorganisms extend into the soil micropores that are not directly available to plant roots and actively assist in phosphate mobilization and uptake	Enhances plant’s access to P from soil	[49,56,72]

**Table 5:** The roles of key microbes involved in the sulfur cycle and their impact on plants.

Microbial Activity	Key Microorganisms	Mechanism of Microbial Activity	Impact on Plants	References
Mineralization	Bacteria (*Bacillus*, *Pseudomonas*, *Clostridium*), fungi (*Aspergillus*, *Penicillium*)	Organic S compounds (e.g., proteins or sulfates) in dead biomass, are broken down by microbial enzymes (e.g., sulfurase or desulfurase) during decomposition	Releases sulfate (SO_4_^2−^), a plant-usable sulfur forms to be absorbed through the roots	[49,56]
Sulfur Oxidation	Bacteria (*Thiobacillus*, *Acidithiobacillus*, *Beggiatoa*, *Sulfolobus*), archae (*Sulfolobus*)	Chemolithotrophic bacteria use reduced sulfur compounds as an energy source, converting them to sulfate; H_2_S → SO_4_^2−^	Convert H_2_S to SO_4_^2−^, enhancing soil S availability	[51,58]
Sulfate Reduction	Bacteria (*Desulfovibrio*, *Desulfobacter*), archae (*Archaeoglobus*)	Sulfate-reducing bacteria (SRBs) use sulfate as an electron acceptor during respiration, producing H_2_S as a byproduct. SO_4_^2−^ → H_2_S	Maintains S cycling in anaerobic soils	[51,58]
Sulfur Immobilization	Soil bacteria, fungi	The organic S compounds are released back into the soil upon microbial death and decomposition	Temporarily stores S in microbial biomass	[6,62]
Mycorrhizal Sulfur uptake	Arbuscular mycorrhizal fungi (*Glomus* species), ectomycorrhizal fungi (*Amanita*, *Boletus*)	Fungal exudation or organic acids (e.g., oxalic acid, citric acid) solubilize bound sulfate in soil. Then, enzymatic activity (e.g., sulfatases) breaks them down into inorganic sulfates (SO_4_^2−^), which are plant-available	Enhances plant access to soil sulfur	[5,75]
Volatilization	Marine phytoplankton and bacteria (*Phaeobacter*, *Roseobacter*)	Certain microbes metabolize S compounds to produce volatile organic S compounds (e.g., dimethyl sulfide) and they can return to soil through precipitation, enriching the S pool	Enriches soil through S deposition from the atmosphere	[6,7,49]

**Table 6:** Buffer margin distance for the spreading of organic fertilizers from waters. * Distance is 10 m for 2 weeks before and 2 weeks after the closed period for spreading organic manure.

Water Body/Number of People Who Use It	Buffer Distance
Water Supply > 100 m^3^ or >500 people	200 m
Water Supply > 10 m^3^ or >50 people	100 m
Water Supply < 10 m^3^ or <50 people	25 m
Lake shoreline	20 m
Exposed cavernous or karstified limestone features	15 m
Any surface watercourse where the slope towards watercourse is >10%	10 m
All other water surfaces *	5 m

## Data Availability

The original contributions presented in this study are included in the article. Further inquiries can be directed to the corresponding author.
